# Profound Modification of Fatty Acid Profile and Endocannabinoid-Related Mediators in PPARα Agonist Fenofibrate-Treated Mice

**DOI:** 10.3390/ijms24010709

**Published:** 2022-12-31

**Authors:** Elisabetta Murru, Anna Lisa Muntoni, Claudia Manca, Sonia Aroni, Marco Pistis, Sebastiano Banni, Gianfranca Carta

**Affiliations:** 1 Department of Biomedical Sciences, University of Cagliari, 09042 Monserrato, Italy; 2 Neuroscience Institute, Section of Cagliari, National Research Council of Italy, 09042 Monserrato, Italy

**Keywords:** fenofibrate, peroxisome proliferator-activated receptor α (PPARα), lipid metabolism, n3-highly unsaturated fatty acid (HUFA) score, endocannabinoids, *N*-acylethanolamines

## Abstract

Fenofibrate (FBR), an oral medication used to treat dyslipidemia, is a ligand of the peroxisome proliferator-activated receptor α (PPARα), a nuclear receptor that regulates the expression of metabolic genes able to control lipid metabolism and food intake. PPARα natural ligands include fatty acids (FA) and FA derivatives such as palmitoylethanolamide (PEA) and oleoylethanolamide (OEA), known to have anti-inflammatory and anorexigenic activities, respectively. We investigated changes in the FA profile and FA derivatives by HPLC and LC-MS in male C57BL/6J mice fed a standard diet with or without 0.2% fenofibrate (0.2% FBR) for 21 days. Induction of PPARα by 0.2% FBR reduced weight gain, food intake, feed efficiency, and liver lipids and induced a profound change in FA metabolism mediated by parallel enhanced mitochondrial and peroxisomal β-oxidation. The former effects led to a steep reduction of essential FA, particularly 18:3n3, with a consequent decrease of the n3-highly unsaturated fatty acids (HUFA) score; the latter effect led to an increase of 16:1n7 and 18:1n9, suggesting enhanced hepatic de novo lipogenesis with increased levels of hepatic PEA and OEA, which may activate a positive feedback and further sustain reductions of body weight, hepatic lipids and feed efficiency.

## 1. Introduction

Fenofibrate (FBR) is a synthetic amphipathic carboxylic acid of the fibrate class, widely used to treat dyslipidemia [1,2,3]. Its biological target is the peroxisome proliferator-activated receptor α (PPARα), a nuclear receptor that acts as a transcription factor to regulate the expression of a plethora of target genes encoding proteins involved in lipid, glucose and amino acid metabolism [4,5]. PPARα maintains lipid homeostasis; it increases lipolysis and activates lipoprotein lipase [4,5,6] and regulates genes involved in fatty acid (FA) uptake and metabolism, mitochondrial and peroxisomal FA oxidation, ketogenesis, triglyceride turnover and gluconeogenesis [5,7]. These effects lead to reduced body weight gain, adiposity, food intake and feed efficiency, as observed in obese rodents treated with dietary FBR [8]. Moreover, PPARα has been shown to possess anti-inflammatory activity [5,9,10].

PPARα is also a sensing receptor for a variety of exogenous nutritional compounds and endogenous metabolites derived from lipid metabolism, such as FA [6] and their endocannabinoid (eCB) derivatives, i.e., 2-arachidonoyl–glycerol (2-AG) and arachidonoylethanolamide (AEA), and eCB-like compounds such as palmitoylethanolamide (PEA) and oleoylethanolamide (OEA), known to have anti-inflammatory and anorexigenic activity, respectively [11,12,13,14]. PPARα natural ligands include dietary n3-polyunsaturated fatty acids (PUFA), such as docosahexaenoic acid (22:6n3, DHA) and eicosapentaenoic acid (20:5n3, EPA) [15], and conjugated linoleic acid (CD18:2, CLA), an FA with a conjugated diene structure naturally present in dairy products [16,17]. In a previous interventional study in humans, we observed that an increase in *PPARα* gene expression following CLA-enriched cheese intake might contribute to the modulation of FA metabolism [16].

Oosterveer et al. showed that treatment of C57Bl/6 mice with FBR increased the expression of gene-encoding enzymes involved in FA synthesis, elongation and desaturation and induced hepatic de novo lipogenesis (DNL) and chain elongation for palmitic (16:0, PA), stearic (18:0, SA) and oleic acid (18:1n9, OA) synthesis [18]. They found that lipogenic induction was supported by sterol regulatory element-binding protein 1c (SREBP-1c) but not by carbohydrate response element-binding protein (ChREBP). Although PPAR and SREBP-1c act in opposite ways in physiological conditions, it has been shown that the presence of *PPARα* is necessary for the proper functioning of Srebp-1c [18,19], which could sustain the induction of stearoyl-coenzyme A desaturase-1 (*Scd1*) upon FBR treatment [18]. FBR treatment simultaneously induced lipogenesis and both peroxisomal and mitochondrial FA β-oxidation, processes that generate acetyl-CoA and NADH, necessary to sustain DNL [18]. 

Based on the recognized ability of FBR to activate PPARα and thus to regulate the expression of a range of metabolic genes able to control lipid metabolism and food intake, we investigated whether changes in tissue FA metabolism triggered by chronic pharmacological activation of PPARα are able to modulate the biosynthesis of their eCB and eCB-like derivatives, which strictly depend on the availability of their FA precursor [20,21] and are involved in the control of food intake and energy expenditure. Furthermore, among eCB-like molecules, PEA and OEA are well-known PPARα ligands [22,23]. 

## 2. Results

### 2.1. Body Weight, Food Intake and Growth

**Figure 1:**
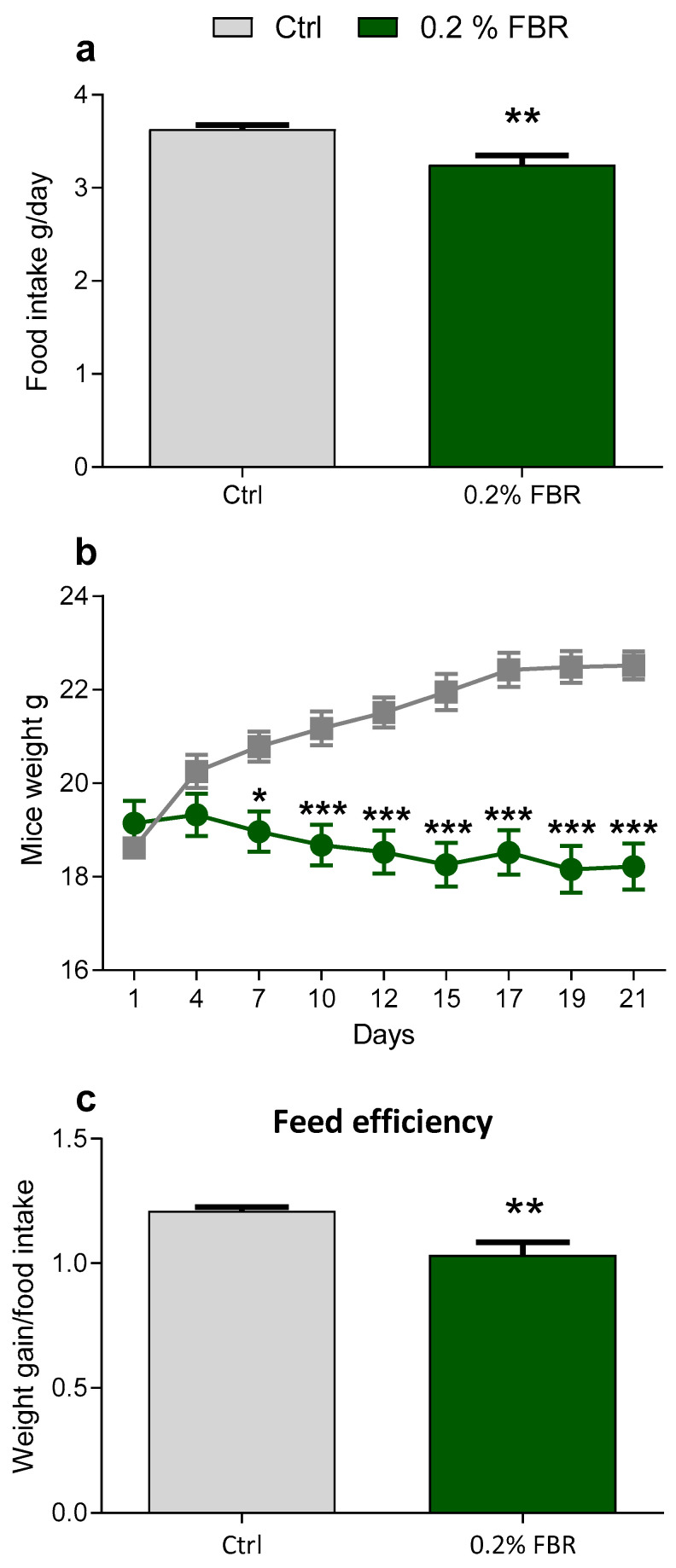
(**a**) Mean food intake (g/day); (**b**) time course of mice weight in g of mice fed a control diet (Ctrl, grey) or a 0.2% FBR-supplemented diet (0.2% FBR, green); (**c**) feed efficiency calculated as percentage of weight gain with respect to time 0 (t0) divided by food intake expressed as percentage of mice fed the control diet. Data are presented as mean ± SEM of n = 10. * *p* ≤ 0.05; ** *p* ≤ 0.01; *** *p* ≤ 0.001.

Mice fed a 0.2% fenofibrate diet (0.2% FBR) for 21 days showed an overall reduction of food intake with respect to mice fed the control diet (Ctrl) (Figure 1a). The weight of 0.2% FBR mice was increasingly reduced from day 7 until day 21, and accordingly their feed efficiency was lower with respect to the control (Figure 1b,c).

### 2.2. Tissue Fatty Acid Profile and eCB-Like Mediators

**Figure 2:**
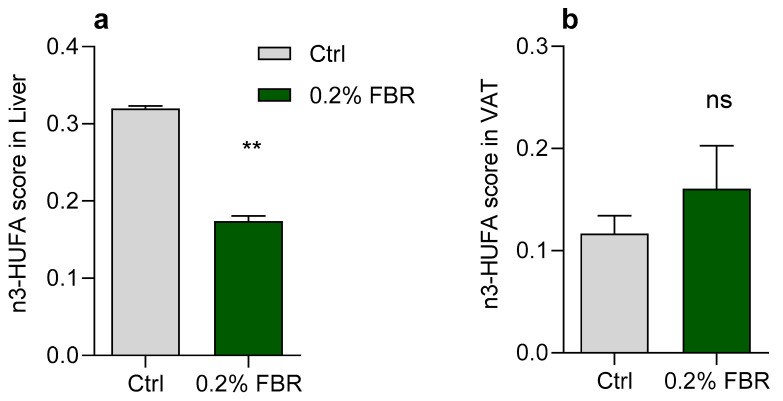
n3-highly unsaturated fatty acid (HUFA) scores of mice fed a control diet (Ctrl) or a 0.2% fenofibrate-supplemented diet (0.2% FBR) in (**a**) liver; (**b**) visceral adipose tissue (VAT). Data are expressed as mean ± SEM of n = 5. ** *p* ≤ 0.01; ns = not significant.

The n3-highly unsaturated fatty acids (HUFA) score was reduced in the liver of 0.2% FBR-treated mice with respect to the control mice, while no changes were observed in visceral adipose tissue (VAT) (Figure 2a,b). 

Hepatic total lipids were lower in 0.2% FBR mice with respect to Ctrl, and this pattern was also observed for n3- and n6-PUFA (Table 1). Arachidonic acid (20:4n6, ARA) and 20:3n6 increased significantly despite the strong reduction of their precursor linoleic acid (18:2n6, LA) and its ∆6 desaturation product γ-linolenic acid (18:3n6, GLA). α-linolenic acid (18:3n3, ALA) was strongly diminished, as was 22:6n3, while 22:5n3 was increased. On the other hand, mead acid (20:3n9, MA) and its precursor 18:1n9 were increased in 0.2% FBR mice, as was the 20:3n9/18:1n9 ratio (Table 1). 

**Figure 3:**
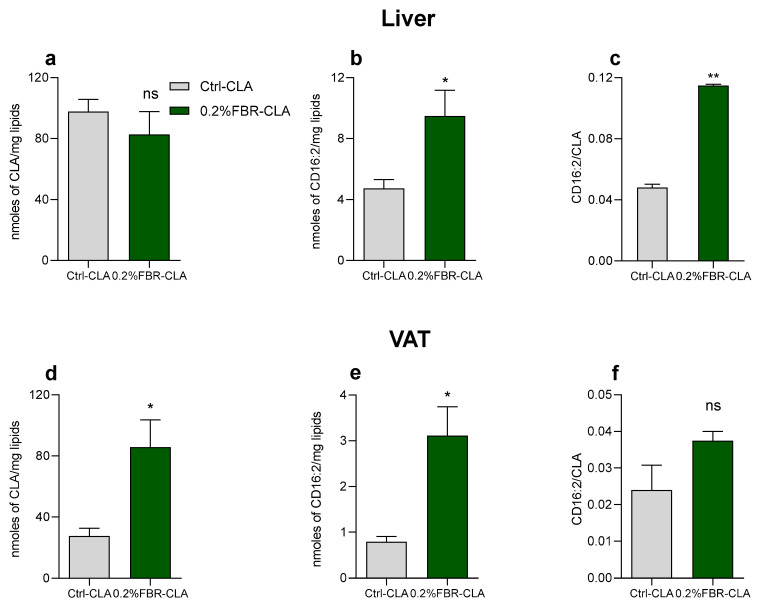
Hepatic levels of (**a**) CLA (CD18:2), (**b**) CD16:2 and (**c**) CD16:2/CLA ratio; visceral adipose tissue (VAT) levels of (**d**) CLA, (**e**) CD16:2 and (**f**) CD16:2/CLA ratio of mice fed a control diet (Ctrl-CLA) or a 0.2% fenofibrate-supplemented diet (0.2% FBR-CLA) treated with a single dose of CLA (90 μg/10 g of body weight by oral gavage). Data are expressed as nmoles/mg of lipids and represent the mean ± SEM of n = 5. * *p* ≤ 0.05, ** *p* ≤ 0.01; ns = not significant.

Acute treatment with the PPARα natural ligand CLA to Ctrl mice (Ctrl-CLA) or 0.2% FBR mice (FBR-CLA) resulted in its increased levels in VAT of 0.2% FBR-CLA mice compared to Ctrl-CLA mice (Figure 3d), while its peroxisomal β-oxidation product, conjugated hexadecadienoic acid (CD16:2), was increased in both liver and VAT, and the CD16:2/CLA ratio was significantly increased in liver (Figure 3b,e,c). 

As shown in Table 1, hepatic 12:0, 14:0 and total saturated FA (SFA) were reduced in 0.2% FBR mice with respect to Ctrl mice, while no changes were found for 16:0 and 18:0. In 0.2% FBR mice, palmitoleic acid (16:1n7, POA), the desaturation product of 16:0, was increased, as was total MUFA; accordingly, there were increased values of the 16:1/16:0 and 18:1/18:0 ratios, considered an expression of ∆9 desaturase activity.

In VAT, levels of 16:1n7, 18:3n3, 18:2n6, and total n3- and n6-PUFA showed the same significant trend as found in liver; no changes were observed for the other FA (Table 2).

**Figure 4:**
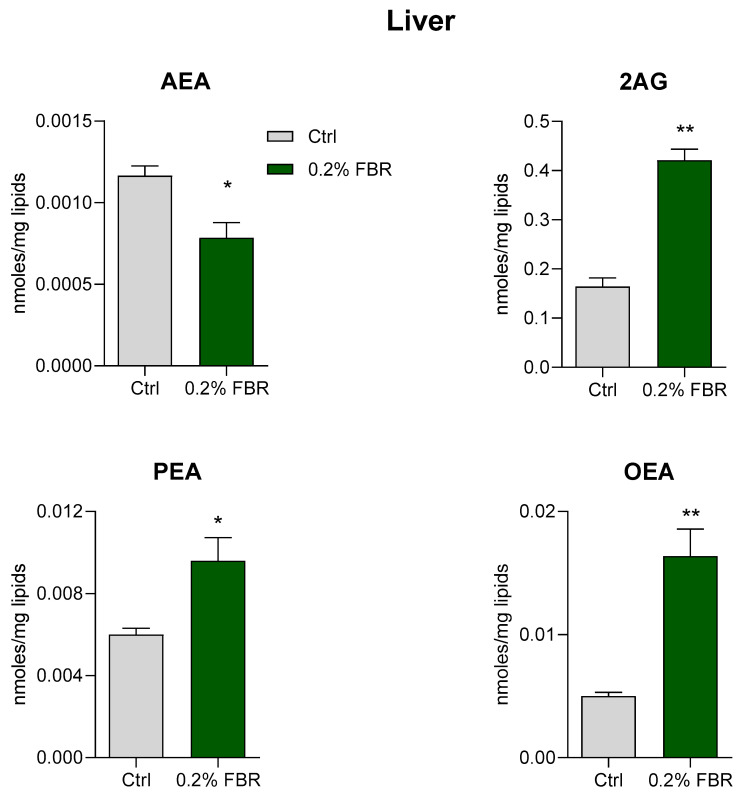
Hepatic levels of the endocannabinoids (eCB), arachidonoylethanolamide (AEA) and 2-arachidonoylglycerol (2-AG), and the eCB-like molecules, palmitoylethanolamide (PEA) and oleoylethanolamide (OEA), in mice fed a control diet (Ctrl) or a 0.2% fenofibrate-supplemented diet (0.2% FBR). Data are expressed as nmoles/mg of lipids and represent the mean ± SEM of n = 5. * *p* ≤ 0.05; ** *p* ≤ 0.01.

**Figure 5:**
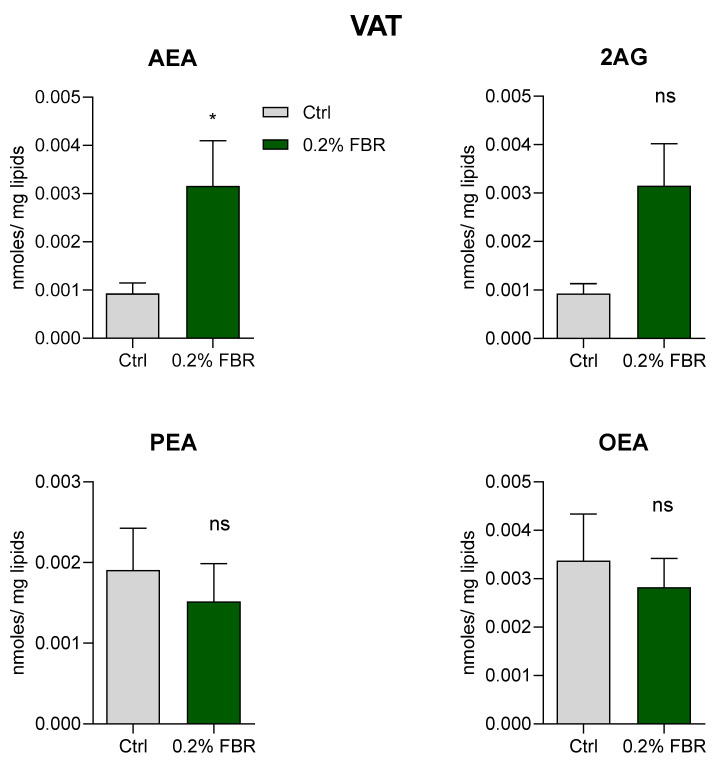
Visceral adipose tissue (VAT) levels of the endocannabinoids (eCB), arachidonoylethanolamide (AEA) and 2-arachidonoylglycerol (2-AG), and the eCB-like molecules, palmitoylethanolamide (PEA) and oleoylethanolamide (OEA), in mice fed a control diet (Ctrl) or a 0.2% fenofibrate-supplemented diet (0.2% FBR). Data are expressed as nmoles/mg of lipids and represent the mean ± SEM of n = 5. * *p* ≤ 0.05; ns = not significant.

The analysis of eCB and eCB-like mediators showed that 0.2% FBR treatment strongly increased OEA, PEA, 2-AG, and decreased AEA levels in liver (Figure 4). In contrast, while AEA was increased in VAT, no other changes were found (Figure 5).

## 3. Discussion

PPARs represent important therapeutic targets for metabolic disorders. PPARα activation by FBR, inducing high rates of mitochondrial and peroxisomal β-oxidation and enhanced lipoproteinlipase activity [24], might decrease the plasma concentrations of triacylglycerol-rich lipoproteins, with a consequent hypotriglyceridemic effect [25,26]. In agreement with other studies showing reduced food intake and body weight following FBR activation of PPARα [27], we observed that chronic (21 days) dietary treatment with 0.2% FBR was able to reduce food intake by 10.5% in mice. Accordingly, body weight was reduced by 9% from day 7, with a progressive, more substantial decrease of up to 19% on day 19. 

Reduced weight gain rather than food intake was the main factor responsible for reduced feed efficiency induced by 0.2% FBR, which may increase energy expenditure [28]. In agreement with this, we also observed a reduction of liver lipids, probably due to enhanced β-oxidation. 

Increased hepatic FA oxidation may strongly influence body weight, as observed in mPPARα-deficient mice, which presented dysfunctional expression of the genes required for FA metabolism in mitochondria and peroxisomes [29,30]. Indeed, we observed in liver, and to some extent in VAT, a strong 7-fold reduction of 18:3n3 and a 2-fold reduction of 18:2n6, precursors of the n3- and n6-PUFA families, respectively. This resulted in an imbalance in the n3/n6 PUFA ratio and a relative reduction of the n3-HUFA score in liver of 0.2% FBR mice. An important consequence of the strong 18:3n3 β-oxidation was the reduction of 22:6n3, albeit less pronounced than expected, probably due to the PPARα-induced enzymes involved in its biosynthesis from 18:3n3, as previously shown [14,18,31]. 

Our data confirm that the massive liver FA β-oxidation induced by the pharmacological activation of PPARα results in a tissue deficiency of essential FA (EFA). This was shown by the increase of 20:3n9, which particularly affects n3-PUFA, since it has been demonstrated that 18:3n3 is the fatty acid preferentially β-oxidized in mitochondria [32]. Therefore, pharmacological activation of PPARα should be co-adjuvated with dietary supplementation of n3-PUFA. On the other hand, by feeding obese rats with CLA, a natural PPARα ligand, we detected an increase in 22:6n3 biosynthesis and thus of the n3-HUFA score in liver [33,34]. Furthermore, in mildly hypercholesterolemic subjects [35] and healthy adults [16], we found that dietary CLA- and 18:3n3-enriched cheese improved the n3-HUFA score in plasma [16]. It is noteworthy that in the study with healthy adults, the higher plasma n3-HUFA score was associated with increased *PPARα* gene expression in leukocytes [16]. 

These contrasting results might be explained by the pharmacological treatment that activates PPARα, possibly inducing mitochondrial β-oxidation that is stronger than the activation of the enzymatic PUFA pathway and peroxisomal β-oxidation that favors 22:6n3 biosynthesis [31], which could instead be sustained by a physiological nutritional treatment.

**Scheme 1:**
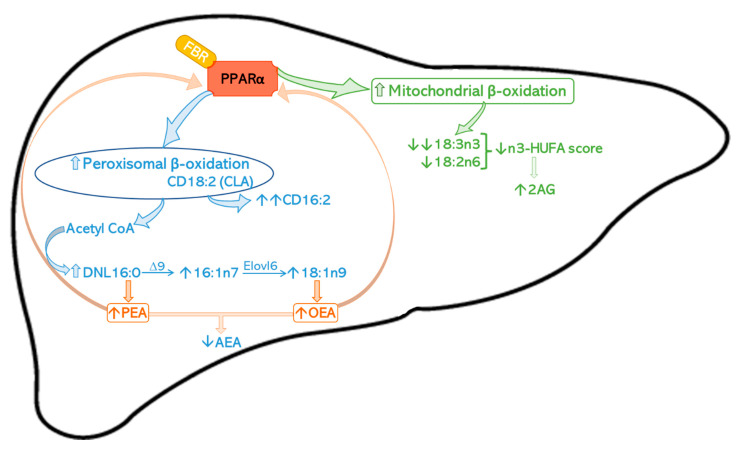
In liver, strong PPARα activation by FBR enhances mitochondrial β-oxidation, particularly of 18:3n3 and to a minor extent of 18:2n6, both preferentially β-oxidized in mitochondria [32]; this leads to a steep reduction of EFA and a decreased n3-HUFA score, which may explain the increase of 2-AG [46,49]. A parallel induction of peroxisomal β-oxidation, confirmed by the elevated formation of CD16:2, the partial peroxisomal β-oxidation product of CD18:2 (CLA) [37], increases acetyl-CoA availability for DNL [18,62,63] and thus the synthesis of 16:0, 16:1n7 and 18:1n9 (its desaturation product by ∆9 desaturase) and further elongation by the PPAR α-induced ELOVL 6 [64]. A rise in hepatic DNL may sustain an increase of hepatic biosynthesis of PEA and OEA, derived from 16:0 and 18:1n9, respectively, known ligands of PPARα and therefore able to further sustain its activation. Reduced hepatic AEA levels in 0.2% FBR-treated mice might be the result of competition in the common biosynthesis pathway with PEA and OEA.

The increase of 16:1n7 and 18:1n9 by 0.2% FBR treatment suggests a rise in DNL in the liver [18,36]. Indeed, it has been proposed that enhanced peroxisomal β-oxidation by 0.2% FBR induces DNL [18]. To evaluate whether peroxisomal β-oxidation was induced in our experimental conditions, we treated mice chronically fed a diet with 0.2% FBR with a single dose of CD18:2 (CLA), which has been shown to be promptly β-oxidized in peroxisomes to CD16:2 [37]. We demonstrated that CD16:2 and the ratio CD16:2/CLA were increased 2-fold in liver, suggesting a strong induction of peroxisomal β-oxidation by FBR in the liver (Scheme 1). Interestingly, in VAT, CLA incorporation was higher in FBR-treated mice, confirming the enhanced lipoprotein lipase activity induced by FBR through activation of PPARα [38].

Several reports have highlighted the ability of eCB-like molecules to bind PPARα, whose activation controls the transcription of enzymes involved in FA metabolism, e.g., elongase and SCD1, and in the metabolism of eCB-like molecules [39,40]; moreover, FBR has been identified as a cannabinoid receptor ligand (CB2) and negative allosteric modulator (CB1) [41]. This led us to investigate whether changes in FA metabolism induced by the PPARα agonist FBR were able to influence the biosynthesis of eCB-like lipid mediators [42]. In addition, we previously showed that in rodent brain slices containing the midbrain, incubated for 1 h either with the synthetic PPARα agonist WY14643 (3 μM) [40] or CLA (100 μM) [43], there was an increase of PEA and OEA levels, which we speculated may further sustain PPARα activity. In the present study we confirmed that PPARα activation in the liver was able to increase the levels of the *N*-acylethanolamines (NAE) PEA and OEA (Scheme 1). Since it has been shown that the biosynthesis of NAE may correlate with the tissue levels of their FA precursors [21], we hypothesize that the enhanced PEA and OEA biosynthesis may derive from a higher availability of their precursors, probably produced by enhanced DNL (Scheme 1). Thus, reduced hepatic AEA levels in 0.2% FBR-treated mice might be the result of competition in the common biosynthesis pathway with PEA and OEA. Such competition does not occur in VAT in which AEA was found to be increased.

2-AG is an eCB and the most abundant of the 2-monoacylglycerols, mirroring the relatively high amounts of 20:4n6 esterified to the sn-2 position of phospholipids from which it derives [44]. Therefore, the dietary FA composition might modulate FA esterified on phospholipids and thus the tissue concentrations of eCB and eCB-like molecules [45]. It has been demonstrated in animal models [46,47,48] and in humans [49] that changes in the n3/n6 PUFA ratio in tissues modulate eCB biosynthesis. Thus, our findings of increased 2-AG in liver and AEA in visceral adipose tissue in 0.2% FBR mice may be explained by the strong reduction of n3-PUFA and of the hepatic n3-HUFA score (Scheme 1).

Changes in eCB and NAE have been described in liver and adipose tissue in the presence of altered lipid and glucose metabolism and inflammation [50,51,52]. An increase in the eCB system tone in peripheral tissues has been shown to inhibit FA oxidation, resulting in a positive energy balance and the development of obesity in mice and humans [53]. Moreover, OEA can decrease hepatic lipid content and serum cholesterol and triglyceride levels through PPARα activation [54]. Therefore, the reduced AEA level and increased OEA and PEA levels in FBR mice found in the present study may support the decreased body weight, reduced hepatic lipids, feed efficiency and inflammation [11,55,56,57]. 

2-AG, similarly to AEA, can be implicated in metabolic disorders as its plasma levels positively correlate with decreased high-density lipoprotein cholesterol and increased triacylglycerol levels and insulin resistance in human studies [58,59]. Moreover, the high 2-AG levels we found in liver of 0.2% FBR mice, being in contrast with changes related to reduced body weight, led us to hypothesize an anti-inflammatory role of this molecule which, despite contrasting results, has been shown to exert anti-inflammatory activity following activation of CB2 in models of acute inflammation [60]. An anti-inflammatory activity of 2-AG could also sustain reduced systemic inflammation following PPARα activation by FBR, as observed in subjects with metabolic syndrome [61].

We conclude that PPARα activation by FBR, by deeply modifying FA metabolism, is able to modulate the biosynthesis of PEA, OEA and the eCB AEA and 2-AG. Since it has been shown that the biosynthesis of these lipid mediators may be modulated by dietary FA, particularly n3-PUFA [49], future studies should evaluate whether n3-HUFA supplementation can further modulate the biosynthesis of eCB and eCB-like molecules, restoring their tissue levels. 

2-arachidonoyl–glycerol (2-AG), arachidonoylethanolamide (AEA), conjugated linoleic acid (CD18:2, CLA), de novo lipogenesis (DNL), elongase 6 (ELOVL 6), essential FA (EFA), fatty acid (FA), fenofibrate (FBR), n3-highly unsaturated fatty acids (HUFA) score, oleoylethanolamide (OEA), peroxisome proliferator-activated receptor (PPAR), palmitoylethanolamide (PEA).

## 4. Material and Methods

### 4.1. Animals and Diets

Male C57BL/6J mice (Harlan, San Pietro al Natisone, Italy) (n = 20; 40 postnatal days) were used. Before the experiments, the mice were housed under a 12 h light–dark cycle (7:00 to 19:00 light), in conditions of constant room temperature (22 °C) and humidity (60%), with food and water ad libitum. After 1 week of acclimation, animals were divided into two experimental groups according to different dietary regimens: (i) a standard rodent diet (Ctrl group, n = 10, 2016 Teklad Global 16% Protein Rodent Diet by Envigo); (ii) a 0.2% *w*/*w* fenofibrate diet (0.2% FBR, n = 10, fenofibrate from Sigma-Aldrich+ 2016 Teklad Global 16% Protein Rodent Diet) [18,65,66,67,68]. The major dietary FA are shown in Table 3.

After 21 days of dietary treatment [67,69], overnight-fasted mice were euthanized. The liver, visceral adipose tissue, frontal cortex and hypothalamus were removed and immediately frozen and stored at −80 °C for further determination if not immediately used. 

**Scheme 2:**
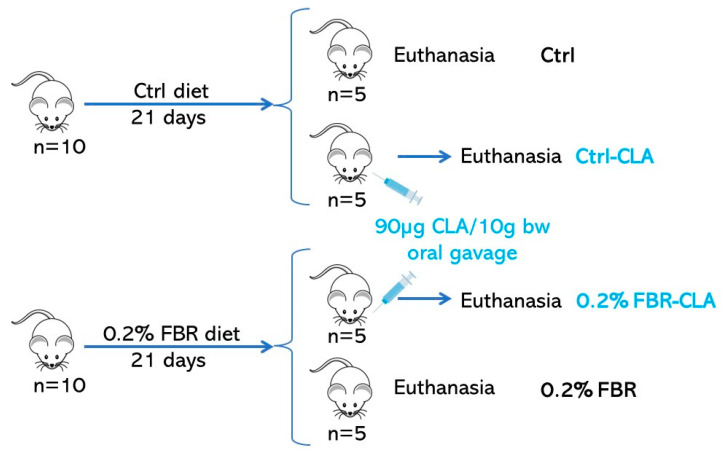
Representation of the experimental design.

To evaluate whether FBR enhanced peroxisomal β-oxidation, a single dose of CLA, previously demonstrated to be promptly β-oxidized in peroxisomes [37], 90 µg/10 g of body weight, was acutely administered by oral gavage to two subgroups of Ctrl and 0.2% FBR mice, Ctrl-CLA (n = 5) and 0.2% FBR-CLA (n = 5), respectively (Scheme 2). CD 16:2, the peroxisomal β-oxidation product of CLA, and the ratio CD16:2/CLA were used as markers of enhanced peroxisomal β-oxidation. 

The weight of the animals was monitored every other day in fasted mice during the experimental period. All study protocols were approved by the Institutional Animal Care and Use Committee.

### 4.2. Lipid Analyses

Total lipids were extracted from tissue samples dissolved in a 2:1 chloroform/methanol solution according to the method of Folch [70] and quantified at a wavelength of 600 nm following the Chiang procedure [71]. All standards and reagents (acetonitrile (CH_3_CN), methanol (CH_3_OH), chloroform (CHCl_3_), n-hexane (C_6_H_14_), ethanol (C_2_H_5_OH), and acetic acid (CH_3_COOH)) were HPLC grade and purchased from Sigma-Aldrich (St. Louis, MO, USA). Ascorbic acid, KOH, deferoxamine mesylate and HCl were purchased from Sigma-Aldrich (St. Louis, MO, USA). Deuterated eCB and related molecules, AEA or anandamide ([^2^H]_8_AEA), 2-AG ([^2^H]_5_2AG), OEA ([^2^H]_2_OEA) and PEA (^2^H]_4_PEA) were purchased from Cayman Chemicals (Ann Arbor, MI, USA).

#### 4.2.1. Measurement of Fatty Acid Composition 

Aliquots of chloroform containing the lipid extract were dissolved in ethanol. Deferoxamine mesylate as iron chelator, a water solution of ascorbic acid as antioxidant, and KOH were then added to mildly saponify at room temperature in order to obtain free fatty acids for HPLC analysis [72] The separation of UFA was carried out using an Agilent 1100 HPLC system (Agilent, Palo Alto, CA, USA) equipped with a diode array detector (DAD). A C-18 Inertsil 5 ODS-2 Chrompack column (Chrompack International BV, Middleburg, The Netherlands) with 5 μm particle size and 150 × 4.6 mm was used with a mobile phase of CH_3_CN/H_2_O/CH_3_COOH (70/30/0.12, *v*/*v*/*v*) at a flow rate of 1.5 mL/min, as previously reported [73]. SFA transparent to UV were measured after methylation as FA methyl esters (FAME) by a gas chromatograph (GC) (Agilent, Model 6890, Palo Alto, CA, USA) equipped with a flame ionization detector (FID) [48].

#### 4.2.2. Quantification of eCB and eCB-Like Molecules

For measurement of the eCB and their related molecules by isotope dilution, deuterated internal standards (AEA ([^2^H]_8_AEA), 2-AG ([^2^H]_5_2AG), OEA ([^2^H]_2_OEA) and PEA (^2^H]_4_PEA)) were previously added to the chloroform/methanol solution [47]. Quantification of AEA, 2-AG, OEA and PEA was carried out by liquid chromatography–atmospheric pressure chemical ionization–mass spectrometry (LC–APCI–MS) using selected ion monitoring (SIM) at M + 1 values for the compounds and their deuterated homologs, as described in [47]. As demonstrated by [74], linearity for quantitative eCB by MS with isotope dilution has been proven over the range of 25 fmol to 250 pmol.

The n3-HUFA score was obtained by calculating the sum of n3 FA with 20 or more carbon atoms and three or more double bonds divided by the sum of total FA with 20 or more carbon atoms and more than two double bonds:

n3-HUFA score = (20:5n3 + 22:6n3 + 22:5n3)/(20:5n3 + 22:6n3 + 22:5n3 + 20:3n6 + 20:4n6 + 22:4n6 + 22:5n6 + 20:3n9) × 100 [75].

Feed efficiency was determined as follows: weight gain/food intake. In detail, weight gain was calculated as percentage of grams of weight gain since time t0, while food intake was expressed as percentage of grams of dietary intake of each mouse with respect to the mean of grams of Ctrl diet intake. 

Calculation of feed efficiency for each mouse either from control or 0.2% FBR groups:

% WG/(g FI × 100/mean g CTRL_FI)

where: WG = percentage of grams of weight gain since time t0; g FI = grams of food intake for each mouse; mean g CTRL_FI = mean of grams of Ctrl dietary intake.

### 4.3. Statistical Analysis

Data are expressed as mean ± SEM. Because the FA, eCB, weight, food intake and feed efficiency data were not normally distributed (Shapiro–Wilk normality test), the statistical significances were assessed using the nonparametric Mann–Whitney test. Multiple unpaired comparison tests were performed by two-way ANOVA followed by the Bonferroni post hoc multiple comparison test for food intake and weight time course. Number of mice per group was calculated using GPower software (G*Power 3.1.9.2). Data were analyzed using the GraphPad Prism 6.01 software (La Jolla, CA, USA) with * *p* ≤ 0.05; ** *p* ≤ 0.01; *** *p* ≤ 0.001 as the cut-off for statistical significance among groups.

## Figures and Tables

**Table 1:** Main fatty acids (FA) in liver of mice fed a control diet (Ctrl) or a 0.2% fenofibrate-supplemented diet (0.2% FBR). Data are expressed as nmoles/mg of lipids and represent the mean ± SEM of n = 5. * *p* ≤ 0.05; ** *p* ≤ 0.01; ns = not significant.

	Ctrl			0.2% FBR			
	Mean		SEM	Mean		SEM	
12:0	3.96	±	0.42	0.75	±	0.03	**
14:0	28.73	±	2.67	10.87	±	0.46	**
16:0	1368.80	±	117.21	1117.72	±	15.18	ns
18:0	353.80	±	45.13	249.95	±	10.58	ns
20:0	5.94	±	0.96	1.79	±	0.34	*
16:1n7	108.82	±	12.44	159.18	±	9.57	*
18:1n9	818.39	±	49.72	1248.86	±	21.48	**
20:3n9	3.63	±	0.09	19.99	±	0.80	**
18:3n3	39.19	±	3.72	5.39	±	0.33	**
20:5n3	6.46	±	0.46	6.24	±	0.75	ns
22:5n3	7.49	±	1.59	14.06	±	1.65	*
22:6n3	191.61	±	11.24	142.41	±	11.06	*
18:2n6	1136.45	±	42.77	568.52	±	12.42	**
18:3n6	40.39	±	3.08	13.04	±	0.60	**
20:2n6	20.33	±	1.52	18.78	±	2.50	ns
20:3n6	25.63	±	1.00	187.26	±	3.69	**
20:4n6	357.35	±	25.03	445.65	±	21.28	*
22:4n6	11.39	±	0.58	12.74	±	0.27	ns
22:5n6	15.05	±	1.09	11.58	±	0.84	ns
SFA ^1^	1778.71	±	160.81	1392.31	±	23.85	*
MUFA ^1^	927.21	±	60.42	1408.03	±	21.77	**
n3-PUFA ^1^	244.76	±	11.41	168.10	±	9.47	**
n6-PUFA ^1^	1606.59	±	61.37	1257.58	±	35.19	**
n3/n6 PUFA ^1^	0.152	±	0.002	0.133	±	0.005	*
16:1n7/16:0	0.080	±	0.008	0.143	±	0.010	**
18:1n9/18:0	2.492	±	0.406	5.032	±	0.233	**
20:3n9/18:1n9	0.005	±	0.000	0.016	±	0.001	**
mg lipids /g tissue	61.84	±	6.44	45.52	±	1.25	**

^1^ saturated FA (SFA), monounsaturated FA (MUFA), polyunsaturated FA (PUFA).

**Table 2:** Main fatty acids (FA) in visceral adipose tissue (VAT) of mice fed a control diet (Ctrl) or a 0.2% fenofibrate-supplemented diet (0.2% FBR). Data are expressed as nmoles/mg of lipids and represent the mean ± SEM of n = 5. ** *p* ≤ 0.01; ns = not significant.

	Ctrl			0.2% FBR			
	Mean		SEM	Mean		SEM	
16:1n7	133.49	±	8.40	305.95	±	30.32	**
18:1n9	1155.96	±	29.38	1163.60	±	45.49	ns
18:3n3	42.34	±	5.66	25.47	±	1.77	**
22:6n3	4.64	±	1.25	5.87	±	1.83	ns
18:2n6	1055.44	±	33.16	640.98	±	30.62	**
18:3n6	5.04	±	0.74	4.83	±	0.81	ns
20:3n6	16.09	±	3.38	12.97	±	1.74	ns
20:4n6	18.63	±	4.97	16.08	±	1.93	ns
MUFA ^1^	1289.45	±	30.22	1469.55	±	69.17	ns
n3-PUFA ^1^	46.97	±	5.88	30.16	±	2.57	**
n6-PUFA ^1^	1095.20	±	36.79	673.89	±	31.22	**
n3/n6 PUFA ^1^	0.043	±	0.006	0.045	±	0.004	ns
mg lipids /g tissue	477.49	±	53.25	362.70	±	60.37	ns

^1^ monounsaturated FA (MUFA), polyunsaturated FA (PUFA).

**Table 3:** Main dietary fatty acid (FA) composition (g/100 g diet) of 2016 Teklad Global control diet as reported by the manufacturer Envigo. Dietary experimental treatments: control diet (Ctrl), control diet + 0.2% fenofibrate (*w*/*w*) (0.2% FBR).

FA	g/100 g
16:0	0.5
18:0	0.1
18:1n9	0.7
18:2n6	2.0
18:3n3	0.1
SFA ^1^	0.6
MUFA ^1^	0.7
PUFA ^1^	2.1

^1^ saturated FA (SFA), monounsaturated FA (MUFA), polyunsaturated FA (PUFA).

## Data Availability

The data presented in the current study are available from the corresponding author upon reasonable request.

## Abbreviations

0.2% fenofibrate diet (0.2% FBR), 2-arachidonoylglycerol (2-AG), arachidonic acid (20:4n6, ARA), arachidonoylethanolamide (AEA), cannabinoid receptor 1 (CB1), carbohydrate response element-binding protein (ChREBP), conjugated linoleic acid (CD18:2, CLA), conjugated hexadecadienoic acid (CD16:2), control diet (Ctrl), de novo lipogenesis (DNL), docosahexaenoic acid (22:6n3, DHA), elongase 6 (ELOVL 6), endocannabinoid (eCB), essential fatty acid (EFA), eicosapentaenoic acid (20:5n3, EPA), fatty acids (FA), fenofibrate (FBR), n3-highly unsaturated fatty acids (HUFA), linoleic acid (18:2n6, LA), α-linolenic acid (18:3n3, ALA), ϒ-linolenic acid (18:3n6, GLA), mead acid (20:3n9, MA), monounsaturated FA (MUFA), *N*-acylethanolamines (NAE), oleic acid (18:1n9, OA), oleoylethanolamide (OEA), palmitic acid (16:0, PA), palmitoleic acid (16:1n7, POA), palmitoylethanolamide (PEA), peroxisome proliferator-activated receptor α (PPARα), polyunsaturated fatty acids (PUFA), saturated FA (SFA), stearoyl-coenzyme A desaturase-1 (∆9 desaturase, SCD1), stearic acid (18:0, SA), sterol regulatory element-binding protein 1c (SREBP-1c), visceral adipose tissue (VAT).

## References

[1] Tenenbaum A., Fisman E.Z. (2012). Fibrates Are an Essential Part of Modern Anti-Dyslipidemic Arsenal: Spotlight on Atherogenic Dyslipidemia and Residual Risk Reduction. Cardiovasc. Diabetol..

[2] McKeage K., Keating G.M. (2011). Fenofibrate: A Review of Its Use in Dyslipidaemia. Drugs.

[3] Nguyen T.N., Park J.-S. (2022). Exploring Fenofibrate Formulations for the Treatment of Lipid Disorders: Past, Present, and Future. Cardiometab. Syndr. J..

[4] Liu A., Patterson A.D., Yang Z., Zhang X., Liu W., Qiu F., Sun H., Krausz K.W., Idle J.R., Gonzalez F.J. (2009). Fenofibrate Metabolism in the Cynomolgus Monkey Using Ultraperformance Liquid Chromatography-Quadrupole Time-of-Flight Mass Spectrometry-Based Metabolomics. Drug Metab. Dispos..

[5] Todisco S., Santarsiero A., Convertini P., De Stefano G., Gilio M., Iacobazzi V., Infantino V. (2022). PPAR Alpha as a Metabolic Modulator of the Liver: Role in the Pathogenesis of Nonalcoholic Steatohepatitis (NASH). Biology.

[6] Bougarne N., Weyers B., Desmet S.J., Deckers J., Ray D.W., Staels B., De Bosscher K. (2018). Molecular Actions of PPARα in Lipid Metabolism and Inflammation. Endocr. Rev..

[7] Patsouris D., Mandard S., Voshol P.J., Escher P., Tan N.S., Havekes L.M., Koenig W., März W., Tafuri S., Wahli W. (2004). PPARα Governs Glycerol Metabolism. J. Clin. Investig..

[8] Larsen P.J., Jensen P.B., Sørensen R.V., Larsen L.K., Vrang N., Wulff E.M., Wassermann K. (2003). Differential Influences of Peroxisome Proliferator–Activated Receptorsγ and -α on Food Intake and Energy Homeostasis. Diabetes.

[9] Chinetti G., Fruchart J.-C., Staels B. (2000). Peroxisome Proliferator-Activated Receptors (PPARs): Nuclear Receptors at the Crossroads between Lipid Metabolism and Inflammation. Inflamm. Res..

[10] Chen H., Charlat O., Tartaglia L.A., Woolf E.A., Weng X., Ellis S.J., Lakey N.D., Culpepper J., More K.J., Breitbart R.E. (1996). Evidence That the Diabetes Gene Encodes the Leptin Receptor: Identification of a Mutation in the Leptin Receptor Gene in Db/Db Mice. Cell.

[11] Borrelli F., Romano B., Petrosino S., Pagano E., Capasso R., Coppola D., Battista G., Orlando P., Di Marzo V., Izzo A.A. (2015). Palmitoylethanolamide, a Naturally Occurring Lipid, Is an Orally Effective Intestinal Anti-Inflammatory Agent: Palmitoylethanolamide and Colitis. Br. J. Pharmacol..

[12] Brown J.D., Karimian Azari E., Ayala J.E. (2017). Oleoylethanolamide: A Fat Ally in the Fight against Obesity. Physiol. Behav..

[13] Campolongo P., Roozendaal B., Trezza V., Cuomo V., Astarita G., Fu J., McGaugh J.L., Piomelli D. (2009). Fat-Induced Satiety Factor Oleoylethanolamide Enhances Memory Consolidation. Proc. Natl. Acad. Sci. USA.

[14] Tahri-Joutey M., Andreoletti P., Surapureddi S., Nasser B., Cherkaoui-Malki M., Latruffe N. (2021). Mechanisms Mediating the Regulation of Peroxisomal Fatty Acid Beta-Oxidation by PPARα. IJMS.

[15] Lamas Bervejillo M., Ferreira A.M., Trostchansky A., Rubbo H. (2019). Understanding Peroxisome Proliferator-Activated Receptors: From the Structure to the Regulatory Actions on Metabolism. Bioactive Lipids in Health and Disease.

[16] Murru E., Carta G., Cordeddu L., Melis M., Desogus E., Ansar H., Chilliard Y., Ferlay A., Stanton C., Coakley M. (2018). Dietary Conjugated Linoleic Acid-Enriched Cheeses Influence the Levels of Circulating n-3 Highly Unsaturated Fatty Acids in Humans. IJMS.

[17] Mollica M.P., Trinchese G., Cavaliere G., De Filippo C., Cocca E., Gaita M., Della-Gatta A., Marano A., Mazzarella G., Bergamo P. (2014). C9,T11-Conjugated Linoleic Acid Ameliorates Steatosis by Modulating Mitochondrial Uncoupling and Nrf2 Pathway. J. Lipid Res..

[18] Oosterveer M.H., Grefhorst A., van Dijk T.H., Havinga R., Staels B., Kuipers F., Groen A.K., Reijngoud D.-J. (2009). Fenofibrate Simultaneously Induces Hepatic Fatty Acid Oxidation, Synthesis, and Elongation in Mice. J. Biol. Chem..

[19] Hebbachi A.M., Knight B.L., Wiggins D., Patel D.D., Gibbons G.F. (2008). Peroxisome Proliferator-Activated Receptor α Deficiency Abolishes the Response of Lipogenic Gene Expression to Re-Feeding. J. Biol. Chem..

[20] Matias I. (2008). Effect of Polyunsaturated Fatty Acids on Endocannabinoid and N-Acyl-Ethanolamine Levels in Mouse Adipocytes. Biochim. Biophys. Acta BBA Mol. Cell Biol. Lipids.

[21] Murru E., Lopes P.A., Carta G., Manca C., Abolghasemi A., Guil-Guerrero J.L., Prates J.A.M., Banni S. (2021). Different Dietary N-3 Polyunsaturated Fatty Acid Formulations Distinctively Modify Tissue Fatty Acid and N-Acylethanolamine Profiles. Nutrients.

[22] Artmann A., Petersen G., Hellgren L.I., Boberg J., Skonberg C., Nellemann C., Hansen S.H., Hansen H.S. (2008). Influence of Dietary Fatty Acids on Endocannabinoid and N-Acylethanolamine Levels in Rat Brain, Liver and Small Intestine. Biochim. Biophys. Acta BBA Mol. Cell Biol. Lipids.

[23] Piomelli D. (2013). A Fatty Gut Feeling. Trends Endocrinol. Metab..

[24] Schoonjans K., Peinado-Onsurbe J., Lefebvre A.M., Heyman R.A., Briggs M., Deeb S., Staels B., Auwerx J. (1996). PPARalpha and PPARgamma Activators Direct a Distinct Tissue-Specific Transcriptional Response via a PPRE in the Lipoprotein Lipase Gene. EMBO J..

[25] Auboeuf D., Rieusset J., Fajas L., Vallier P., Frering V., Riou J.P., Staels B., Auwerx J., Laville M., Vidal H. (1997). Tissue Distribution and Quantification of the Expression of MRNAs of Peroxisome Proliferator–Activated Receptors and Liver X Receptor-α in Humans: No Alteration in Adipose Tissue of Obese and NIDDM Patients. Diabetes.

[26] Mukherjee R., Jow L., Croston G.E., Paterniti J.R. (1997). Identification, Characterization, and Tissue Distribution of Human Peroxisome Proliferator-Activated Receptor (PPAR) Isoforms PPARγ2 versus PPARγ1 and Activation with Retinoid X Receptor Agonists and Antagonists. J. Biol. Chem..

[27] Park M.-K., Han Y., Kim M.S., Seo E., Kang S., Park S.-Y., Koh H., Kim D.K., Lee H.-J. (2012). Reduction of Food Intake by Fenofibrate Is Associated with Cholecystokinin Release in Long-Evans Tokushima Rats. Korean J. Physiol. Pharmacol..

[28] Mancini F.P., Lanni A., Sabatino L., Moreno M., Giannino A., Contaldo F., Colantuoni V., Goglia F. (2001). Fenofibrate Prevents and Reduces Body Weight Gain and Adiposity in Diet-Induced Obese Rats. FEBS Lett..

[29] Lee S.S., Pineau T., Drago J., Lee E.J., Owens J.W., Kroetz D.L., Fernandez-Salguero P.M., Westphal H., Gonzalez F.J. (1995). Targeted Disruption of the Alpha Isoform of the Peroxisome Proliferator-Activated Receptor Gene in Mice Results in Abolishment of the Pleiotropic Effects of Peroxisome Proliferators. Mol. Cell. Biol..

[30] Costet P., Legendre C., Moré J., Edgar A., Galtier P., Pineau T. (1998). Peroxisome Proliferator-Activated Receptor α-Isoform Deficiency Leads to Progressive Dyslipidemia with Sexually Dimorphic Obesity and Steatosis. J. Biol. Chem..

[31] Ferdinandusse S., Denis S., Mooijer P.A.W., Zhang Z., Reddy J.K., Spector A.A., Wanders R.J.A. (2001). Identification of the Peroxisomal β-Oxidation Enzymes Involved in the Biosynthesis of Docosahexaenoic Acid. J. Lipid Res..

[32] Burdge G.C. (2006). Metabolism of α-Linolenic Acid in Humans. Prostaglandins Leukot. Essent. Fat. Acids.

[33] Carta G., Murru E., Manca C., Serra A., Mele M., Banni S. (2019). Natural CLA-Enriched Lamb Meat Fat Modifies Tissue Fatty Acid Profile and Increases n-3 HUFA Score in Obese Zucker Rats. Biomolecules.

[34] Piras A., Carta G., Murru E., Lopes P.A., Martins S.V., Prates J.A.M., Banni S. (2015). Effects of Dietary CLA on N-3 HUFA Score and N-Acylethanolamides Biosynthesis in the Liver of Obese Zucker Rats. Prostaglandins Leukot. Essent. Fat. Acids.

[35] Pintus S., Murru E., Carta G., Cordeddu L., Batetta B., Accossu S., Pistis D., Uda S., Elena Ghiani M., Mele M. (2013). Sheep Cheese Naturally Enriched in α-Linolenic, Conjugated Linoleic and Vaccenic Acids Improves the Lipid Profile and Reduces Anandamide in the Plasma of Hypercholesterolaemic Subjects. Br. J. Nutr..

[36] Ito Y., Hudgins L.C., Hirsch J., Shike M. (1991). Adipose-Tissue Fatty Acid Composition in Recipients of Long-Term Total Parenteral Nutrition (TPN). Am. J. Clin. Nutr..

[37] Banni S., Petroni A., Blasevich M., Carta G., Angioni E., Murru E., Day B.W., Melis M.P., Spada S., Ip C. (2004). Detection of Conjugated C16 PUFAs in Rat Tissues as Possible Partial Beta-Oxidation Products of Naturally Occurring Conjugated Linoleic Acid and Its Metabolites. Biochim. Biophys. Acta BBA Mol. Cell Biol. Lipids.

[38] Heller F., Harvengt C. (1983). Effects of Clofibrate, Bezafibrate, Fenofibrate and Probucol on Plasma Lipolytic Enzymes in Normolipaemic Subjects. Eur. J. Clin. Pharmacol..

[39] Iannotti F.A., Vitale R.M. (2021). The Endocannabinoid System and PPARs: Focus on Their Signalling Crosstalk, Action and Transcriptional Regulation. Cells.

[40] Melis M., Carta G., Pistis M., Banni S. (2013). Physiological Role of Peroxisome Proliferator-Activated Receptors Type Alpha on Dopamine Systems. CNSNDDT.

[41] Priestley R.S., Nickolls S.A., Alexander S.P.H., Kendall D.A. (2015). A Potential Role for Cannabinoid Receptors in the Therapeutic Action of Fenofibrate. FASEB J..

[42] Stasiulewicz A., Znajdek K., Grudzień M., Pawiński T., Sulkowska J.I. (2020). A Guide to Targeting the Endocannabinoid System in Drug Design. IJMS.

[43] Murru E., Carta G., Manca C., Sogos V., Pistis M., Melis M., Banni S. (2021). Conjugated Linoleic Acid and Brain Metabolism: A Possible Anti-Neuroinflammatory Role Mediated by PPARα Activation. Front. Pharmacol..

[44] Lands W.E.M. (2000). Stories about Acyl Chains. Biochim. Biophys. Acta BBA Mol. Cell Biol. Lipids.

[45] Bisogno T., Maccarrone M. (2014). Endocannabinoid Signaling and Its Regulation by Nutrients: Endocannabinoid Signaling and Nutrients. BioFactors.

[46] Di Marzo V., Griinari M., Carta G., Murru E., Ligresti A., Cordeddu L., Giordano E., Bisogno T., Collu M., Batetta B. (2010). Dietary Krill Oil Increases Docosahexaenoic Acid and Reduces 2-Arachidonoylglycerol but Not N-Acylethanolamine Levels in the Brain of Obese Zucker Rats. Int. Dairy J..

[47] Piscitelli F., Carta G., Bisogno T., Murru E., Cordeddu L., Berge K., Tandy S., Cohn J.S., Griinari M., Banni S. (2011). Effect of Dietary Krill Oil Supplementation on the Endocannabinoidome of Metabolically Relevant Tissues from High-Fat-Fed Mice. Nutr. Metab..

[48] Batetta B., Griinari M., Carta G., Murru E., Ligresti A., Cordeddu L., Giordano E., Sanna F., Bisogno T., Uda S. (2009). Endocannabinoids May Mediate the Ability of (n-3) Fatty Acids to Reduce Ectopic Fat and Inflammatory Mediators in Obese Zucker Rats. J. Nutr..

[49] Banni S., Carta G., Murru E., Cordeddu L., Giordano E., Sirigu A., Berge K., Vik H., Maki K.C., Di Marzo V. (2011). Krill Oil Significantly Decreases 2-Arachidonoylglycerol Plasma Levels in Obese Subjects. Nutr. Metab..

[50] Veilleux A., Di Marzo V., Silvestri C. (2019). The Expanded Endocannabinoid System/Endocannabinoidome as a Potential Target for Treating Diabetes Mellitus. Curr. Diab. Rep..

[51] Gatta-Cherifi B., Cota D. (2016). New Insights on the Role of the Endocannabinoid System in the Regulation of Energy Balance. Int. J. Obes..

[52] Muccioli G.G., Naslain D., Bäckhed F., Reigstad C.S., Lambert D.M., Delzenne N.M., Cani P.D. (2010). The Endocannabinoid System Links Gut Microbiota to Adipogenesis. Mol. Syst. Biol..

[53] Kuipers E.N., Kantae V., Maarse B.C.E., van den Berg S.M., van Eenige R., Nahon K.J., Reifel-Miller A., Coskun T., de Winther M.P.J., Lutgens E. (2019). High Fat Diet Increases Circulating Endocannabinoids Accompanied by Increased Synthesis Enzymes in Adipose Tissue. Front. Physiol..

[54] Fu J., Oveisi F., Gaetani S., Lin E., Piomelli D. (2005). Oleoylethanolamide, an Endogenous PPAR-α Agonist, Lowers Body Weight and Hyperlipidemia in Obese Rats. Neuropharmacology.

[55] Diep T.A., Madsen A.N., Holst B., Kristiansen M.M., Wellner N., Hansen S.H., Hansen H.S. (2011). Dietary Fat Decreases Intestinal Levels of the Anorectic Lipids through a Fat Sensor. FASEB J..

[56] Rodríguez de Fonseca F., Navarro M., Gómez R., Escuredo L., Nava F., Fu J., Murillo-Rodríguez E., Giuffrida A., LoVerme J., Gaetani S. (2001). An Anorexic Lipid Mediator Regulated by Feeding. Nature.

[57] Thabuis C., Destaillats F., Landrier J.-F., Tissot-Favre D., Martin J.-C. (2010). Analysis of Gene Expression Pattern Reveals Potential Targets of Dietary Oleoylethanolamide in Reducing Body Fat Gain in C3H Mice. J. Nutr. Biochem..

[58] Blüher M., Engeli S., Klöting N., Berndt J., Fasshauer M., Bátkai S., Pacher P., Schön M.R., Jordan J., Stumvoll M. (2006). Dysregulation of the Peripheral and Adipose Tissue Endocannabinoid System in Human Abdominal Obesity. Diabetes.

[59] Côté M., Matias I., Lemieux I., Petrosino S., Alméras N., Després J.-P., Di Marzo V. (2007). Circulating Endocannabinoid Levels, Abdominal Adiposity and Related Cardiometabolic Risk Factors in Obese Men. Int. J. Obes..

[60] Parlar A., Arslan S., Doğan M., Ayşenur Çam S., Yalçin A., Elibol E., Kaya Özer M., Üçkardeş F., Kara H. (2018). The Exogenous Administration of CB2 Specific Agonist, GW405833, Inhibits Inflammation by Reducing Cytokine Production and Oxidative Stress. Exp. Ther. Med..

[61] Belfort R., Berria R., Cornell J., Cusi K. (2010). Fenofibrate Reduces Systemic Inflammation Markers Independent of Its Effects on Lipid and Glucose Metabolism in Patients with the Metabolic Syndrome. J. Clin. Endocrinol. Metab..

[62] Burdge G.C., Wootton S.A. (2003). Conversion of α-Linolenic Acid to Palmitic, Palmitoleic, Stearic and Oleic Acids in Men and Women. Prostaglandins Leukot. Essent. Fat. Acids.

[63] Sinclair A.J. (1975). Incorporation of Radioactive Polyunsaturated Fatty Acids into Liver and Brain of Developing Rat. Lipids.

[64] Wang Y., Botolin D., Christian B., Busik J., Xu J., Jump D.B. (2005). Tissue-Specific, Nutritional, and Developmental Regulation of Rat Fatty Acid Elongases. J. Lipid Res..

[65] Puligheddu M., Melis M., Pillolla G., Milioli G., Parrino L., Terzano G.M., Aroni S., Sagheddu C., Marrosu F., Pistis M. (2017). Rationale for an Adjunctive Therapy with Fenofibrate in Pharmacoresistant Nocturnal Frontal Lobe Epilepsy. Epilepsia.

[66] Puligheddu M., Pillolla G., Melis M., Lecca S., Marrosu F., De Montis M.G., Scheggi S., Carta G., Murru E., Aroni S. (2013). PPAR-Alpha Agonists as Novel Antiepileptic Drugs: Preclinical Findings. PLoS ONE.

[67] Li J., Bi L., Hulke M., Li T. (2014). Fish Oil and Fenofibrate Prevented Phosphorylation-Dependent Hepatic Sortilin 1 Degradation in Western Diet-Fed Mice. J. Biol. Chem..

[68] Toyoda T., Kamei Y., Kato H., Sugita S., Takeya M., Suganami T., Ogawa Y. (2008). Effect of Peroxisome Proliferator-Activated Receptor-α Ligands in the Interaction Between Adipocytes and Macrophages in Obese Adipose Tissue. Obesity.

[69] Caillaud M., Patel N.H., Toma W., White A., Thompson D., Mann J., Tran T.H., Roberts J.L., Poklis J.L., Bigbee J.W. (2020). A Fenofibrate Diet Prevents Paclitaxel-Induced Peripheral Neuropathy in Mice. Cancers.

[70] Folch J., Lees M., Sloane Stanley G.H. (1957). A Simple Method for the Isolation and Purification of Total Lipides from Animal Tissues. J. Biol. Chem..

[71] Chiang S.P., Gessert C.F., Lowry O.H. (1957). Colorimetric Determination of Extracted Lipids. An Adaptation for Microgram Amounts of Lipids Obtained from Cerumen. Curr. List Med. Lit. Res. Rep..

[72] Banni S., Carta G., Contini M.S., Angioni E., Deiana M., Dessì M.A., Melis M.P., Corongiu F.P. (1996). Characterization of Conjugated Diene Fatty Acids in Milk, Dairy Products, and Lamb Tissues. J. Nutr. Biochem..

[73] Melis M.P., Angioni E., Carta G., Murru E., Scanu P., Spada S., Banni S. (2001). Characterization of Conjugated Linoleic Acid and Its Metabolites by RP-HPLC with Diode Array Detector. Eur. J. Lipid Sci. Technol..

[74] Richardson D., Ortori C.A., Chapman V., Kendall D.A., Barrett D.A. (2007). Quantitative Profiling of Endocannabinoids and Related Compounds in Rat Brain Using Liquid Chromatography–Tandem Electrospray Ionization Mass Spectrometry. Anal. Biochem..

[75] Stark K.D. (2008). The Percentage of N-3 Highly Unsaturated Fatty Acids in Total HUFA as a Biomarker for Omega-3 Fatty Acid Status in Tissues. Lipids.

